# The prevalence of ocular *Demodex folliculorum* in 2253 young males

**DOI:** 10.1038/s41598-022-26782-y

**Published:** 2022-12-26

**Authors:** Qian Ye, Weiming Yan, Yunpeng Wang, Meizhu Chen

**Affiliations:** Department of Ophthalmology, The 900th Hospital of Joint Logistic Support Force, PLA (Clinical Medical College of Fujian Medical University, Dongfang Hospital Affiliated to Xiamen University), Fuzhou, 350025 China

**Keywords:** Conjunctival diseases, Eyelid diseases

## Abstract

To investigate the prevalence and influencing factors of *Demodex folliculorum* (DF) in the eyelashes of healthy young males. An epidemiological cross-sectional prevalence study was conducted. We conducted visual acuity, eye-related examination, eyelash microscopic examination and DF count of recruits in Fujian Province in 2019. The presence of DF was analyzed according to age, keratorefractive surgery, annual household income, educational level, long-term residence, sleep time, time of using electronic products, smoking and drinking habit. A total of 2253 healthy young males (aged 17–24 years) were studied for the presence of DF within eyelash follicles. The total prevalence of DF was 20.73% in our study. Subjects with history of keratorefractive surgery had a statistically significant increase in the prevalence of DF (*P* < 0.001). The prevalence of DF was higher in subjects with ocular symptoms than in those without symptoms (*P* < 0.05). Factors such as the educational level and so on we analyzed had no significant correlation with the prevalence of DF (all *P* > 0.05). According to the multivariate logistic regression analysis, the history of keratorefractive surgery was the risk factors of DF infection (*P* < 0.001), and the risk of infection was 1.437 times higher in the population with the history of keratorefractive surgery than in the population without. There was no correlation between ocular discomfort and DF infection (*P* > 0.05). The prevalence of DF in eyelash follicles in healthy young males was relatively high. The history of keratorefractive surgery was an important risk factor for the infection.

## Introduction

*Demodex* belong to the *Arachnid*, *Acarids*, and *Demodicidae* of the *Arthropoda*^1^. *Demodex folliculorum* (DF) and *Demodex brevis* are the only two that^2^ could live in human body and reside especially in the sebaceous glands in the facial skin, such as the nose, nasolabial folds, eyelids, cheek, forehead, chin and neck^3,4^. In the eye, DF is primarily found in clusters around the eyelash root and the eyelash follicles. *Demodex brevis* resides solitarily in the meibomian and sebaceous glands around the eyelash follicles^5^. A growing body of evidences indicate that DF plays an important role in the onset of many ocular surface diseases, such as blepharitis^6^, allergic conjunctivitis^7^, pterygium^8^, chalazia^9^, and periocular basal cell carcinoma^10^. Misdiagnosis of DF infection during these diseases might lead to failure of treatment, relapse and even serious complications^11^. Patients with ocular DF infection often complain of dry eyes, burning eyes, foreign body sensation, photophobia, increased secretions and repeated eyelash loss^12^. The symptoms in refractory blepharitis patients were significantly improved after anti-DF treatment. Meanwhile, DF could also appears in healthy people. Investigators propose that DF has symbiotic relationship with humans and is beneficial as it ingests bacteria in the follicular canal^13^. It was reported that the prevalence of DF infection increases with age^14^. The positive rate of the population at age 60 years was showed to be 84%, and 100% in those older than 70 years^15^. Infection in children under 10 years of age is rare^16^. Some study reported the presence of DF in children with immunodeficiency and in leukemia patients^17^. However, the available literature presents no data on the prevalence of ocular DF in young males. *Demodex* are acquired and become abundant during puberty because of theirs high production of secretions by sebaceous gland, which may create optimal conditions for DF infection and reproduction^18^. We speculated that the young males may be a higher risk group in respect of DF infection.

Based on that assumption we conducted a prospective cross-sectional survey to investigate the prevalence of DF infection in the eyelashes of healthy young males. Besides, the relationship between the ocular symptoms, lifestyle habits and DF infection in this particular group were also evaluated.

## Materials and methods

### Subject data

The prospective cross-sectional survey was conducted with a total of 2287 recruits (aged 17–25 years) in Fujian Province. They came from all over the country, and had passed the initial physical examination for enlistment. The enlistment physical examination standard included: a height more than 160 cm; a weight not more than 30% of the standard weight and not less than 15% of the standard; no heart diseases, hypertension and other systemic diseases; no communicable diseases and mental diseases. The enlistment ophthalmic standards included: a visual acuity that was 20/50 or above in the right eye, with 20/60 or above in the left eye. The best corrected visual acuity (BCVA) of both eyes was 20/30 or above. If myopia correction surgery was done, it could only be keratorefractive surgery, and the time was more than 6 months. The history was reported negative for any ocular diseases and any other ocular surgery. They were assigned for the re-examination. All subjects signed a consent document to participate in our study.

### Investigation content

Each subject was required to fill in a questionnaire, which included the basic information, such as the age, the educational level, the long-term residence, the annual household income, the sleep time, the time of using electronic products, the smoking and the drinking habits, and the ocular symptoms (Table 1). The visual acuity was acquired using the International Standard Vision Chart. The eye condition, especially the eyelid margin, the eyelashes, and the conjunctiva, was examined under the slit-lamp microscope (SLM-1ER; KangHua, Chongqing, China). Subjects with ocular discomfort, such as burning, itching, and redness, were examined more carefully to see whether there was a presence of blepharitis or not. The clinical signs included eyelashes with cylindrical dandruff, palpebral margin hyperaemia and hypertrophy, irritation of the eyelids, disorders of the eyelashes and so on.

**Table 1:**
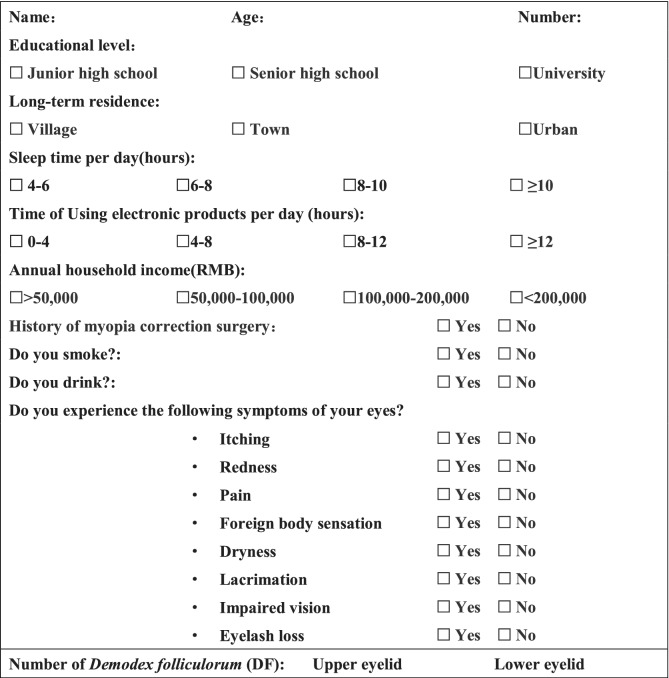
Questionnaire.

For each subject, a total of 6 eyelashes were collected by a sterile forceps. In great details, three eyelashes from the middle position of both the upper and lower eyelids of the left eye were collected. They were placed in parallel on the slide and covered with a slip, respectively. Eyelashes from each subject were observed one by one to count the number of DF under an optical microscope (XSP-H1600; AOSVI, Shenzhen, China). Specially, the number of DF on the eyelashes of both the upper and the lower eyelid was confirmed three times without error. Furthermore, the presence of DF in any one of the eyelashes was also defined as DF-positive. The results were also registered in the above questionnaire, with the number of DF counted under microscopy.

### Quality control

The survey was performed by 15 clinically-experienced ophthalmologists. A chief ophthalmologist, the project leader, was in charge of the survey. The project leader and all the team members had an epidemiological survey experience, and had undergone unified training to be proficient in the operation. Each questionnaire was checked whether it was completely filled or not. All the collected data were reviewed three times by the other investigator after the survey.

### Statistical analysis

The survey data were collected by a special-assigned person. Data were analyzed using a commercially-available statistical software package (SPSS for windows, Version 24.0; IBM-SPSS, Chicago, IL, US). The prevalence of DF and its influencing factors were analyzed by the Chi-square test and the logistic regression analysis. A *P* value less than 0.05 was considered statistically significant.

### Ethics approval and consent to participate

The Ethics Committee of the 900th Hospital of Joint Logistic Support Force, PLA approved the publication of this article, and participants had provided informed consents for publication of this work.

## Results

### General data

Of all the 2287 recruits, 2253 (98.51%) completed the questionnaire. The other 34 recruits did not completely fill out the questionnaire (Table 1) and were excluded. All the 2253 subjects were males, with a mean age of 19.61 ± 1.52 years. The DF was found in 467 subjects, with an overall prevalence of 20.73% (467/2253).

### Presence of DF in different eyelids

The prevalence of DF in the upper and lower eyelid was 13.18% (297/2253) and 10.47% (236/2253), respectively. Among them, 2.93% (66/2253) of the subjects showed DF-positive in both the upper and lower eyelids. The prevalence of DF in the upper eyelid was higher than that of the lower eyelid, and the difference was statistically significant (*P* < 0.001). Of the 467 positive subjects, a total of 846 DF were detected, with 501 in the upper eyelid and 345 in the lower eyelid. The number of DF in one subject ranged from 1 to 9. The mean number of DF was 0.30 per eyelash of each positive subject, with 0.35 per eyelash in the upper eyelid and 0.24 in per eyelash of the lower eyelid (Table 2).

**Table 2:** The *Demodex folliculorum* (DF) prevalence of the upper and lower eyelids [number].

The upper eyelid	The lower eyelid		Total	χ^2^	*P*
	DF-positive	DF-negative			
DF-positive	66	231	297	50.342	< 0.001
DF-negative	170	1786	1956		
Total	236	2017	2253		

### The relationship of DF presence with various factors

Sequence of the prevalence of DF in different ages from the highest to the lowest was below: 23–24, 19–20, 17–18, 21–22. The DF prevalence in subjects with the educational level of university diploma was higher than that of senior high school, but lower than that of junior high school. The DF prevalence in subjects living in village, town and urban was 19.75%, 22.37% and 21.25%, respectively. Subjects with household annual income of > 200,000 RMB had a highest DF prevalence of 28.57%, and subjects with household annual income of 100,000–200,000 RMB had a lowest DF prevalence of 18.86%. And subjects with household annual income of < 50,000 RMB, 50,000–100,000 RMB had a similar DF prevalence of 20.65%, 20.55%, respectively. No statistical difference regarding the DF prevalence was found among different age, educational levels, long-term residences or annual household income (all *P* > 0.05).

Subjects using the electronic products 8–12 h per day had the highest infection rate among 4 groups of different time spent on using electronic products. However, the difference was not statistically significant with the others (*P* > 0.05). The DF prevalence in subjects with the sleep time of more than 10 h per day was the highest compared to subjects with the other sleep time. No significant difference was found between the sleep time and the presence of DF (*P* > 0.05). The DF prevalence of smokers was lower than that of non-smokers. Conversely, subjects who drank had a higher DF prevalence than those who denied drinking. No Statistically significant relationship was found between the presence of DF and the habits of smoking and drinking (all *P* > 0.05).

Of all the surveyed 2253 subjects, 698 had undergone keratorefractive surgery and 25.07% (175/698) were found DF*-*positive. 18.78% of those patients (292/1555) who denied the history of keratorefractive surgery were DF*-*positive. The prevalence of DF was significantly higher in the subjects with a history of keratorefractive surgery (*P* < 0.001). (Table 3).

**Table 3:** Distribution of *Demodex folliculorum* (DF) in relation to studied variables [number (%)].

Variable	DF-positive	DF-negative	Total
**Age**			
17–18	125 (20.70)	479 (79.30)	604 (100.00)
19–20	231 (21.77)	830 (78.23)	1061 (100.00)
21–22	84 (17.54)	395 (82.46)	479 (100.00)
23–24	27 (24.77)	82 (75.23)	109 (100.00)
χ^2^ = 4.754, *P* = 0.190			
**Educational level**			
Junior high school	108 (23.08)	360 (76.92)	468 (100.00)
Senior high school	288 (19.77)	1169 (80.23)	1457 (100.00)
University diploma	71 (21.65)	257 (78.35)	328 (100.00)
χ^2^ = 2.559, *P* = 0.278			
**Long-term residence**			
Village	256 (19.75)	1040 (80.25)	1296 (100.00)
Town	153 (22.37)	531 (77.63)	684 (100.00)
Urban	58 (21.25)	215 (78.75)	273 (100.00)
χ2 = 1.914, *P* = 0.384			
**Household annual income**			
> 50.000	192 (20.65)	738 (79.35)	930 (100.00)
50.000–100.000	194 (20.55)	750 (79.45)	944 (100.00)
100.000–200.000	53 (18.86)	228 (81.14)	281 (100.00)
< 200.000	28 (28.57)	70 (71.43)	98 (100.00)
χ^2^ = 4.287, *P* = 0.232			
**Time of using electronic products (hour)**			
0–4	233 (21.22)	865 (78.78)	1098 (100.00)
4–8	182 (19.59)	747 (80.41)	929 (100.00)
8–12	47 (24.35)	146 (75.65)	193 (100.00)
≥ 12	5 (15.15)	28 (84.85)	33 (100.00)
χ^2^ = 3.060, *P* = 0.382			
**Sleep time (hour)**			
4–6	4 (18.18)	18 (81.82)	22 (100.00)
6–8	165 (23.11)	549 (76.89)	714 (100.00)
8–10	279 (19.36)	1162 (80.64)	1441 (100.00)
≥ 10	19 (25.00)	57 (75.00)	76 (100.00)
χ^2^ = 5.032, *P* = 0.169			
**Surgery**			
Yes	175 (25.07)	523 (74.93)	698 (100.00)
No	292 (18.78)	1263 (81.22)	1555 (100.00)
χ^2^ = 11.613, *P* < 0.001			
**Smoke**			
Yes	142 (20.23)	560 (79.77)	702 (100.00)
No	325 (20.95)	1226 (79.05)	1551 (100.00)
χ^2^ = 0.155, *P* = 0.694			
**Drinking**			
Yes	75 (24.51)	231 (75.49)	306 (100.00)
No	392 (20.13)	1555 (79.87)	1947 (100.00)
χ^2^ = 3.082, *P* = 0.079			
**Total**	**467**	**1786**	**2253**

### The relationship of DF presence with ocular symptoms

Generally, 20.55% of all the surveyed subjects reported the presence of at least one ocular symptom among itching, redness, pain, foreign body sensation, dryness, watery, blurred vision and eyelash loss. And none of them had a presence of blepharitis. The remaining 79.45% subjects did not have any ocular symptoms. A total of 199 DF were detected in the all the symptomatic subjects, while 647 DF were found in the asymptomatic ones. Specifically, the prevalence pf DF in the symptomatic subjects (24.19%, 112/463), was significantly higher than that in the asymptomatic subjects (19.83%, 355/1790) (*P* < 0.05). The average number of DF in one eyelash for both the symptomatic and asymptomatic groups was 0.30 per eyelash. (Table 4).

**Table 4:** Ocular symptoms in *Demodex folliculorum* (DF) positive and negative subjects [number].

Symptom	DF-positive	DF-negative	Total	χ^2^	*P*
Yes	112	351	463	5.189	0.023
No	355	1435	1790		
Total	467	1786	2253		

Among the above studied factors, keratorefractive surgery history and the ocular symptoms were found to be statistically associated with DF-presence by the one-single logistic analysis (both *P* < 0.05). With the multivariate logistic regression analysis, the history of keratorefractive surgery was the risk factor of DF infection while the ocular discomfort was not. The risk in the population with the history of keratorefractive surgery was 1.437 times higher than those without the history of surgery. (Table 5).

**Table 5:** The multivariate regression analysis.

Variable	B	S.E	OR	95% CI	*P*
Surgery	0.363	0.109	1.437	1.161–1.780	< 0.001
Symptom	0.241	0.124	1.271	0.998–1.623	0.052

## Discussion

As far as we know, no similar study has been conducted on such a large group of healthy subjects for investigating the DF prevalence. Biernat et al. found a DF prevalence of 24.3% (28/115) in healthy volunteers^19^. DF was found in 26.7% (88/330) of healthy control group by Kemal et al.^20^. A single-center study from India reported a DF prevalence of 18.0% in healthy subjects^6^. Zhong et al.^21^ revealed a DF prevalence of 8.47% in healthy subjects. Meanwhile, a DF prevalence of 54.9% (28/51) in healthy subjects was found in Kabataş’s study^22^. The prevalence of DF in different studies was varied. The followings may be the underlying reasons. Firstly, the number of eyelashes taken from each subject was different among studies ranging from 4 to 12. Kabataş took only 2 eyelashes from the upper and lower eyelids of one eye with a total of 4 eyelashes for detection of DF^22^. In Zhong’s study, 2 eyelashes were removed from each eyelid, with a total of 8 eyelashes prepared for DF detection^21^. Kemal epilated 3 eyelashes from each eyelid, with a total of 12 eyelashes for study^20^. In Biernat’s study, a sample of 10 eyelashes was taken randomly from every subject^19^. Theoretically, the more eyelashes taken for detection, the higher prevalence of DF is. In fact, the detected DF prevalence was not significantly increased with the number of eyelashes. Meanwhile, the discomfort of the surveyed subjects increased. Therefore, we took 3 eyelashes from the upper eyelid and 3 from the lower eyelid to get a better compliance from the subjects and to follow the Chinese consensus recommendation^23^. Secondly, the location of eyelashes taken from the eyelid was inconsistent among studies. The upper eyelid has a greater eyelash density and a deeper hair follicle, which is speculated to be more suitable for DF’s colonization and reproduction. We confirmed the hypothesis as the prevalence and number of DF in upper eyelids from our result were higher than those in lower eyelids and the same result was seen in our other study^24^. Thirdly, the subjects we investigated were all male, while other studies had different ratios of male to female. Biernat and Kemal reported that no association of *Demodex* infection with gender was found^19,20^. Zhong found that the prevalence of *Demodex folliculorum* was higher in females than in males due to the application of exogenous lipidsin cosmetics^21^. Forthly, it’s not specified the eye from which the eyelashes were plucked out in the other studies we refer to. Kheirkhah reported that *Demodex* infection occured in both eyes^25^. And Xing et al. reported that there was no statistical difference in the chance of *Demodex* infection between the left and right eyes^26^. Because the number of subjects was large and the staff was right-handed, we chose the left eye for eyelash extraction. Whether Ocular *Demodex* infection is symmetrically in both eyes or not need further research.

Although subjects with ocular symptom had a higher prevalence of DF in our study, no correlation between ocular symptom and the prevalence of DF was revealed by the multivariate logistic regression analysis. Our findings were in accordance with the study by Wesolowska et al.^27^. However, other studies found a positive relationship between the presence of DF and the ocular symptom^28,29^. In great details, Rodríguez et al. found a greater incidence of DF in the blepharitis patients who had obvious ocular symptoms^30^. The number of subjects and the DF detection levels might account for the difference among various studies on the relationship between ocular symptom and DF infection. In our study, 698 subjects had received keratorefractive surgery at least 6 months ago, the keratorefractive surgery they received may affect the ocular symptom. Due to atypical symptoms of DF infection, the description of the symptoms was not quantified in our current study. Further study would be conducted with the ocular surface disease index (OSDI) score.

Due to the visual acuity requirements for recruits and the increase of adolescent myopia, 30.98% subjects had undergone keratorefractive surgery. Our study found a higher DF prevalence in the subjects with keratorefractive surgery*,* which was confirmed as a risk factor by further logistic regression analysis. According to our knowledge, association between the keratorefractive surgery and presence of DF has not been studied so far. The keratorefractive surgery could result in damage to the corneal nerve, which could cause the neurotrophic keratopathy^31^. Besides, the conjunctival goblet cells might also be damaged leading to decreased mucin secretion^32^. This would further lead to the decrease of tear secretion and tear film stability. In addition, the use of antibiotic eyedrops during the perioperative period could also cause the flora imbalance. It could further push the DF to move out from the bottom of the follicle to the palpebral margin. Furthermore, the use of postoperative oculentum is easy to cause the accumulation of secretion at the root of the eyelashes. All the above factors would disorder the ocular surface microenvironment after keratorefractive surgery, making it more prone for the DF infection. What’s more, Nearsighted people have the possibility to wear contact lenses, which may affect the presence of DF. The type of myopia correction surgery implicated for DF infestation can only be specific to keratorefractive surgery which’s the standard of the examination for enlistment, and many of subject didn’t know more specific operational styles.

The DF is mainly transmitted through direct body contact and is related to poor sanitary conditions^27^.Our result revealed that the annual household income, the education level and the long-term residence had no effect on the DF infection. The sleep time, the time of using electronic products, the habits of smoking and drinking were also not related with the DF infection, which might probably due to the good hygiene habits of subjects. Subjects using the electronic products 8–12 h per day had a relatively high DF prevalence. Prolonged electronic use may be more susceptible to eye strain, myopia and myopia correction surgery, therefore DF infect. Wesolowska reported that there were no differences in the DF prevalence between people living in old houses and those living in newer ones, which was somewhat in consistence with our result^27^. However, Kabataş found a significant relationship between the habits of smoking and drinking with the DF infection^22^. The subjects in our study were all recruit, concealment of bad habits may lead to the difference with that of Kabataş. In addition, the items in questionnaire such as annual household income, sleep time, time of using electronic products were variable. Different grouping criteria may have different results. The use of eyedrops may be a influencing factors of DF -positive, which we didn’t include in our questionnairs. Our next step is to optimize our questionnaire and make it more reliable.

The prevalence of DF between different ages in our study were not significantly different, which might due to the small age range of the subjects. Meanwhile, the age of our subjects mainly ranged in 17–24 year-old. Our results could help add to the data of DF prevalence in this special group of people.

In conclusion, our study revealed a relatively high prevalence of ocular DF in healthy young males. The history of keratorefractive surgery was an important risk factor for the DF infection, while no correlation between DF infestation and ocular symptom was presented. Further studies are needed to gain better insight into the relationship between ocular symptoms and DF infection in young people.

## Data Availability

The data that support the findings of this study are available from the corresponding author upon reasonable request. The dataset used in this study analysis is not currently available.
